# Evolving Trends and Future Demands in ENT Procedures: A Nationwide 10-Year Analysis

**DOI:** 10.3390/jcm13247850

**Published:** 2024-12-23

**Authors:** Akash Jangan, Satvir Minhas, Emmanuel Diakos, Mark Simmons, Zahir Mughal

**Affiliations:** 1 Department of ENT, Walsall Healthcare NHS Trust, Moat Rd., Walsall WS2 9PS, UK; 2 Department of ENT, Oxford University Hospitals NHS Foundation Trust, Headley Way, Oxford OX3 9DU, UK

**Keywords:** Hospital Episodes Statistics (HES), workforce planning, otolaryngology, otology, rhinology, head and neck

## Abstract

**Objective**: This study aims to investigate the trends in otology, rhinology, and head and neck (H&N) operations over the past decade in England. These trends will allow for predictive modelling to forecast the demand over the coming years to aid workforce and resource planning in ENT. **Methods**: Hospital Episode Statistics data were extracted between April 2012 and April 2023. A total of 121 otology, 114 rhinology, and 122 H&N procedure codes were included. Correlation and linear regression analyses were conducted to examine trends and produce a forecast model for the volume of operations. **Results**: A gradual upward trend in the volume of operations was observed in rhinology, with a positive correlation coefficient (R = 0.74). In contrast, otology (R = −0.67) and H&N (R= −0.75) showed negative trends, indicating a moderate decline in operational volumes over time. The COVID-19 pandemic significantly disrupted operating activity in rhinology and otology. To address the backlog and reach the pre-pandemic forecasted levels within the next five years, surgical capacity must increase by an additional 33,807 rhinology 25,486 otology, and 10,300 head procedures per year in England. **Conclusions**: This analysis highlights a need for prioritization and expansion of the ENT workforce and resources to manage the current backlog and anticipated increase in demand over the next five years.

## 1. Introduction

Ear, Nose, and Throat (ENT) surgery encompasses a diverse range of conditions, including those affecting hearing, balance, breathing, swallowing, voice, and head and neck (H&N) cancers. Over time, the landscape of ENT procedures has undergone significant changes, influenced by rapid technological advancements, shifting patient demographics, evolving patterns of disease burden, and the impact of healthcare policies. These dynamic factors present considerable challenges for the National Health Service (NHS) in the United Kingdom, which is already under significant strain due to increasing demands and stretched resources.

Technological advancements, such as endoscopic sinus surgery, cochlear implants, laser techniques, robotic surgery, and the use of artificial intelligence, are constantly changing the field of otolaryngology, enhancing the precision and scope of surgical procedures [1,2,3,4,5]. However, these innovations have also introduced a level of unpredictability to the demand for specific procedures, requiring flexible and forward-thinking approaches to resource planning. Additionally, demographic shifts, such as the aging population in the UK, are contributing to changes in the prevalence of ENT conditions. For instance, the number of people aged over 85 is projected to grow by 55% by 2037 [6]. This is expected to lead to an increased prevalence of chronic conditions such as hearing loss, H&N cancers, and chronic rhinosinusitis. Therefore, it is likely to intensify the demand for otology, rhinology, and H&N surgical services, necessitating strategic planning to meet future healthcare needs.

The Coronavirus Disease 2019 (COVID-19) pandemic has further compounded the challenges faced by the NHS, profoundly disrupting healthcare delivery worldwide. In the UK, elective surgical waiting lists grew from 4.4 million before the pandemic to 6 million in 2022, with an estimated 10 million patients delaying or forgoing treatment altogether [7]. This unprecedented backlog has placed additional pressure on an already-overstretched healthcare system. Although the NHS Constitution mandates that 92% of patients should wait no longer than 18 weeks from referral to treatment, this standard has not been achieved since 2015 [8]. The 2022 delivery plan for tackling the elective care backlog highlighted the difficulty in predicting how quickly services could recover, reinforcing the urgent need for improved resource allocation and workforce optimization.

Effective workforce planning is essential for ensuring the sustainability of ENT services and optimizing healthcare delivery. The UK surgical workforce census published in 2023 emphasized that the current workload is unsustainable for the existing workforce, underscoring the importance of understanding trends in procedural demand to anticipate and address future challenges [9]. The NHS believes that workforce planning requires aligning the right people with the appropriate skills in the right locations at the right time to deliver the highest standard of patient care [6]. To achieve this, a detailed analysis of trends in procedural volumes within surgical specialties is key to guiding resource allocation and workforce development.

Despite the clinical and practical importance of procedural analyses, there remains a paucity of detailed studies examining trends in ENT procedures. Hospital Episode Statistics (HES) data provide a comprehensive dataset covering procedures performed in all NHS hospitals across England from 2000 to 2023 [10]. This publicly available resource offers a unique opportunity to analyze inpatient and outpatient activity on a national scale, providing valuable insights into trends and procedural volumes [10]. Using this dataset, this study investigates trends in otology, rhinology, and H&N procedures over the past decade to inform resource planning and workforce optimization. There is a specific focus on the impact of COVID-19 on pre-pandemic operating volumes in ENT. The goal is to ensure the NHS is equipped to meet the evolving healthcare needs of its population, to guide the pathway to recovery post-COVID-19, and to support the development of sustainable ENT services for the future.

## 2. Materials and Methods

### 2.1. Data Collection

HES data was accessed through online records on the NHS Digital website. This data included the number of patients undergoing surgical procedures in each fiscal year from April 2012 to April 2023. Procedures were entered into a Microsoft Excel spreadsheet with their specific Office of Population Censuses and Surveys (OPCS-4) codes. A total of 121 otology, 114 rhinology, and 122 H&N procedure codes were included, as listed in Supplementary File S1. The HES data from 2000–2001 to 2011–2012 was excluded from the trend analysis as there was an arbitrary shift in procedure volumes across all subspecialties between the years 2011–2012 and 2012–2013 (Supplementary File S2). This increase is not accounted for but may be related to changes in reporting practices. Therefore, in order to provide the most accurate and up-to-date analysis, only the time period beyond 2012–2013 was included in this study.

Data corresponding to the COVID-19 period (2020–2021 to 2022–2023) was excluded from trend analysis as this period was an anomaly. The fiscal year April 2020–April 2021 was selected as the beginning of the COVID-19 period because the first UK-wide lockdown was announced in March 2020 and the NHS restructured its services and workforce, severely impacting elective activity [11]. The pre-COVID-19 (2019–2020) year volumes were compared to the operative volumes in each subsequent year, and this was presented as a percentage reduction. To further assess the impact of COVID-19, we estimated the backlog created by the pandemic. First, we calculated the difference between the forecasted number of operations during the COVID-19 period and the actual number of operations during this period. This comparison enabled us to determine the annual deficit. Then, by summing these annual deficits, we estimated the cumulative backlog of operations generated by the pandemic. Finally, to propose a strategy for clearing the pandemic backlog, we calculated the additional number of operations needed for each subspeciality to clear the backlog over the next five years.

### 2.2. Statistical Tests

The statistical tests were performed on the total volume of ENT procedures (rhinology, otology and H&N) as well as each individual subspecialty. Correlation analysis was performed to assess the linear relationship between time and number of operations. The correlation coefficient (R) was calculated to quantify the direction and strength of the linear relationship. Linear regression analysis was conducted to evaluate the regression model and its predictive power. Coefficient of determination (R^2^) was reported to evaluate the goodness of fit of the regression model. Our analysis was based on the data from 2012–2013 to 2019–2020. All analyses were performed using Python 3.11. This study involved secondary analysis of anonymized HES data, and, therefore, no ethical approval was required according to the NHS Health Research Authority online tool.

## 3. Results

### 3.1. Trend Analysis

**Figure 1:**
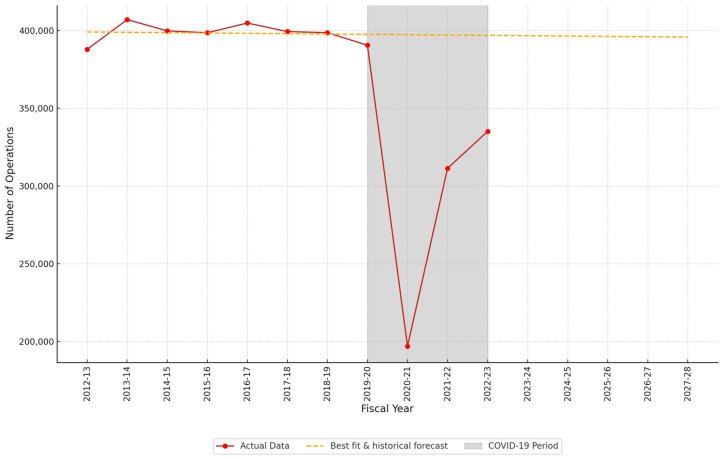
Trend analysis of cumulative ENT procedures. The line of best fit represents the linear regression trend based on pre-pandemic (2012–2013 to 2019–2020) volumes with a five-year forecast regression line. Grey area = COVID-19 period. Fiscal year = April to April.

**Figure 2:**
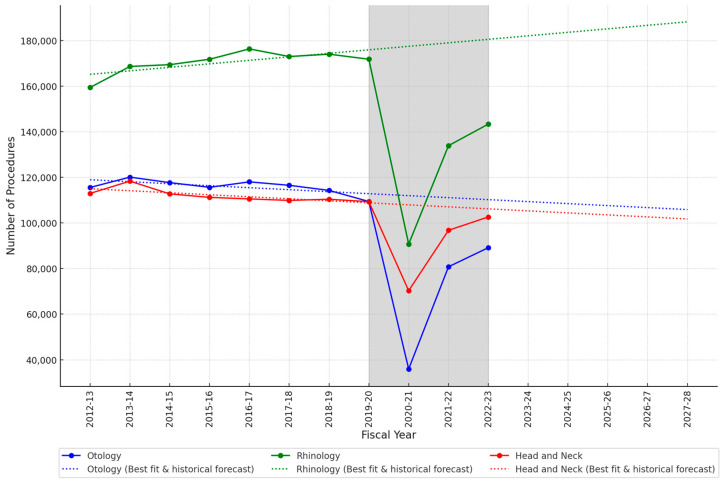
Trend analysis of ENT subspecialties with five-year forecast regression lines for each subspecialty based on pre-COVID-19 data. Grey area = COVID-19 period. Fiscal year = April to April.

The trend for the total ENT procedure volumes across all subspecialties showed that pre-pandemic (2012–2013 to 2019–2020) volumes were generally stable, with no significant fluctuations (Figure 1). The correlation coefficient was −0.08, indicating a very weak correlation. The trend of ENT procedures across the three subspecialties over time is shown in Figure 2. Rhinology showed a positive correlation coefficient (R = 0.74), indicating a strong upward trend in the number of operations. However, H&N (R = −0.75) and otology (R = −0.67) showed negative trends, reflecting moderate declines in operating numbers during the analyzed period.

### 3.2. Forecast Analysis

The coefficient of determination (R^2^) values quantified the proportion of variance in procedure volumes that could be explained by the linear regression model. The R^2^ was 0.007 for the cumulative number of ENT procedure volumes, suggesting that time alone explained only a very small portion of the variation in procedure volumes, likely due to other influencing factors. The R^2^ values were rhinology 0.54, H&N 0.56, and otology 0.45. These values indicated a moderate linear trend over time, implying the model captured the trend reasonably well.

### 3.3. Impact of COVID-19

The pandemic caused significant disruption to operating activity, as depicted by a sharp deviation from the predicted trends, highlighted in grey shade in both Figure 1 and Figure 2. Compared to pre-pandemic levels, in 2020–2021, there was an overall 50.7% decrease in the total number of ENT procedures performed (Figure 1). This improved to a 22.0% decrease in 2021–2022 and 16.1% decrease in 2022–2023 (Figure 1).

The volume of operations in the most recent recorded year, 2022–2023, showed near-complete recovery in H&N, but procedure volumes in otology and especially rhinology remained below the predicted pre-pandemic forecasted levels for 2022–2023 (Figure 2).

The estimated cumulative backlog of operations created by the pandemic was significant, with approximately 169,034 operations in rhinology, 127,431 in otology, and 51,499 operations in H&N (Table 1).

### 3.4. Strategy for the Next Five Years

To address the backlog created by the pandemic within the next five years, by 2027–2028, 33,807 procedures in rhinology, 25,486 in otology, and 10,300 in H&N are needed in addition to new cases.

## 4. Discussion

### 4.1. Main Findings

The findings show that, nationally, across England, the overall ENT procedure volumes remained stable between April 2012 and April 2020, suggesting that demand for ENT services was relatively constant during this pre-pandemic period. Interestingly, however, whilst overall trends were stable, amongst the subspecialities, an upward trend was observed in rhinology and downward trends in otology and H&N procedures. A scoping review of the literature suggests that this study is the most comprehensive analysis of the nationwide ENT subspecialty procedure volumes during the pre-pandemic and pandemic period. The total number of ENT procedures in this study was stable over a decade; in contrast, globally, multiple single-center studies report an increase in ENT operating, but this may be more reflective of local practices as opposed to national demand [12,13]. Institutional practices and local expertise often influence the choice and adoption of medical procedures. For instance, a recent single-center study advocates incorporating sliding epiglottoplasty as an additional step for pharyngeal reconstruction following laryngectomy, rather than relying solely on primary closure. This approach not only affects procedural volumes but also underscores the role of institutional preferences in shaping clinical practices [14]. Similarly, the National Institute for Health and Care Excellence (NICE) issued guidance on the use of Hypoglossal Nerve Stimulation for moderate to severe obstructive sleep apnea (OSA) in 2017 [15]. Despite this, the first UK center to perform this procedure was Guy’s and St Thomas’ Hospital in London in 2023 [16]. This demonstrates how geographical location, and local expertise can significantly influence the availability and adoption of new procedures, leading to disparities in procedural volumes.

The upward trend in rhinology is likely multifactorial and influenced by changes in technology, adoption of new techniques, and evolving management guidelines and policies. For instance, an increase in endoscopic sinus surgery has been observed in multiple previous studies [1,17,18]. This may be attributable to advancements in technologies and increasing popularity and application of endoscopic techniques, which are minimally invasive. Similarly, there has been a significant development in the usage of laser technologies in rhinology [19]. Additionally, the positive trend in rhinology may be inflated by the coding of multiple procedures performed during a single session. For example, endoscopic sinus surgeries often encompass multiple individual procedures, such as septoplasty or turbinate surgery. Therefore, a single operative encounter may be coded under multiple procedures, which could account for the relatively increased number of rhinology procedures over H&N and otology. Furthermore, in recent years, there has been a change in the management guidelines of certain conditions, such as chronic rhinosinusitis (CRS), with a push for early surgical intervention [20]. This shift in patient management may be contributing to the increasing demand for rhinology procedures. Similarly, a recent randomized controlled trial recommended septoplasty as a more effective intervention than medical management for nasal obstruction associated with a deviated nasal septum [21]. This recommendation is likely to drive future demand for these procedures for functional indications. Patient expectations and behavior may have evolved with time and patients may increasingly seek surgical options for chronic conditions affecting quality of life, such as obstructive nasal airflow and snoring.

A downwards trend was observed in otology and H&N procedures. This may be attributable to changes in the clinical management of chronic conditions. Additionally, the downwards trend in H&N can be explained by advances in non-surgical alternatives to managing malignancies, such as radiotherapy, chemotherapy and biological therapies. Comparably, in France, the overall number of ENT cancer surgeries declined between 2010 and 2021, with a sharper decrease observed during the COVID-19 pandemic [22]. There have been limited studies which looked at nationwide data on otological or H&N procedure volumes. However, trends in specific procedures within each subspecialty have been reported. A national study in Scotland reported a decline in ventilation tube insertion in children between 2001 and 2018 [23]. Finland and the US report similar declines in tympanostomies [24,25]. Similarly, a nationwide South Korean study shows a reduction in cholesteatoma surgery between 2012 and 2018 [26]. These individual studies are comparable to our findings for otology. In contrast, several studies report that total thyroidectomies, a common H&N procedure, appears to have increased in the United States of America (US) over a similar period [27,28].

### 4.2. Impact of COVID-19

The findings suggest that, compared to 2019–2020, there was an approximately 50.7% reduction in elective ENT procedures in the year 2020–2021 and this has gradually improved but has not reached pre-pandemic levels yet. These findings are comparable to other studies in the literature. In the US, similar trends have been seen in ENT as well as other specialties, including cardiology, orthopedics, and ophthalmology [29,30,31,32]. A single-center UK study conducted in 2021 showed a 31.2% reduction in ENT surgical procedures post-COVID-19, and despite low volumes of COVID-19 infection, the operating levels have not returned to baseline [33]. Similarly, our study shows that operating volumes in 2022–2023 are only 84% of the levels in 2019–2020. This may be secondary to changes in elective practices as new safety protocols have reduced the efficiency and speed at which elective surgery is administered, suggesting that, nationally, we are yet to return to pre-pandemic operating levels, let alone clear the backlog generated by the pandemic.

Considering the subspeciality data, a relatively small difference in pre- and post-COVID-19 forecasts was observed in H&N procedures. The data show that rhinology and otology were more severely impacted by COVID-19 compared to H&N. This difference might be due to the nature of these procedures, many of which are urgent or oncological and, thus, less susceptible to deferral or cancellation compared to more elective or non-urgent procedures in rhinology and otology.

There is an urgent need for strategic expansion and optimization of operational capacity to manage the anticipated increase in demand for new cases and address the backlog of deferred treatments. Our analysis indicates that, to clear the backlog within the next five years, the volume of operations per year must increase by an additional 10,300 in H&N, 25,468 in otology, and 33,807 in rhinology. This significant upscaling in surgical capacity is essential to address the current backlog while meeting the ongoing demand for ENT surgery.

### 4.3. Application of Findings and Recommendations

This study can be used to provide several recommendations for workforce planning. ENT departments should prioritize addressing the significant variations in backlog and demand across the specialties. Based on these data, rhinology has the largest backlog as well as the strongest upward trend in demand. This suggests that a significant investment in additional workforce, training, and theatre allocation is needed in this subspecialty. The five-year timeline for recovery suggested by our forecast analysis is an example of how our study can be used to set a clearance target nationally. These data may be used to advocate for national policy changes to prioritize specialties like rhinology, which are facing high demand and backlogs. Furthermore, they can be used to advocate for targeted funding programs to accelerate recovery.

Our study highlights the need for integrating predictive models into national healthcare strategies to proactively allocate resources appropriately, to plan for future demands. It is apparent that although a linear regression model does give a foundational idea of basic trends, it does not fully account for the multiple cofounding variables that have been discussed. Furthermore, we recommend the integration of real-time analytics into hospital systems to provide more accurate forecasting for future procedural demands. Transparency and availability of these data to the general population can aid in public awareness about the backlog and its impact in order to help manage patient expectations for delayed procedures.

Training and workforce development are key areas for translating the findings of this study. This study highlights the need for targeted workforce training programs in rhinology. For instance, additional fellowship programs may address the high demand for procedures by upskilling surgeons in emerging techniques and recruiting additional specialists in departments. Alternatively, H&N and otology, whilst experiencing a moderate backlog, should focus on sustaining procedure volumes and address challenges related to the declining pre-pandemic trend. This may suggest that more focus and attention are needed in the outpatient workload management of these subspecialties, as this may help to identify patients who may benefit from surgery. Insights into the trends in outpatient workload will be extremely valuable.

In addition, these data can help provide a framework for scheduling activity within departments. For instance, the data show that the volume ratio of otology/rhinology/H&N in the year 2022–2023 was approximately equal to 1:2:1. These proportions can be utilized to dictate how theatre time is divided between subspecialities. Similarly, they can influence the proportion of subspecialists that are recruited into a department. These data can also aid in prioritizing cases for waiting list initiatives such as additional operating theatre sessions scheduled outside of regular contractual hours. A potential partnership with the private healthcare providers that can perform low-risk, high-volume procedures may aid in freeing up NHS resources for more complex cases.

### 4.4. Limitations

This study relied on retrospective HES data, using an administrative dataset, and, therefore, may not fully capture the breadth of clinical practice. The reporting and coding of procedures are likely to evolve with technologies, practice, and computer software within the NHS. For example, the Royal College of Physicians, in collaboration with the Department of Health, aimed to improve the quality and accuracy of clinical coding through the iLab project [34]. While these improvements are beneficial, they can introduce arbitrary shifts in data over extended periods of time, leading to measurement bias and complicating trend analysis. We observed a notable unexplained shift in reporting trends between the first and second decade of the 20-year span of HES data, as illustrated in Supplementary File S2. Consequently, we used the latter segment of data to ensure the most up-to-date and accurate data analysis.

The predictive modelling provided an estimate of the anticipated volume of operations. However, it was based on pre-COVID-19 activity levels and, therefore, assumes that in the post-COVID-19 period, the rate of trend will be the same. With only a few years of post-COVID-19 data available, these data were volatile and had to be excluded from the trend analysis. The R^2^ values for the three subspecialties were moderate, suggesting that a linear model does not fully account for unexamined factors influencing the observed trends. For instance, a report by the Financial Times highlighted a record surge in private healthcare demand in 2023 [35]. This trend is largely attributed to public dissatisfaction with NHS waiting times and resource constraints. As a result, our forecast model may overestimate the pandemic-induced backlog, as a portion of these cases may have already been addressed by the private sector.

A key limitation of predictive modelling is its inability to account for unforeseen changes in future healthcare delivery innovations, or policy shifts that could impact on future clinical activity. For example, while not currently funded by the NHS, biologic therapy has the potential to reduce the need for repeated endoscopic sinus surgery for CRS with nasal polyps in the future [36]. Conversely, in otology, the expanding criterion for cochlear implantation is likely to increase the demand for this procedure [37]. Such advancements could alter the demand for certain surgical procedures.

### 4.5. Future Research

Trend analysis of national data provides valuable insights into overall service demand but may not be generalizable to individual ENT departments. Resource availability, patient demographics, and clinical practices may vary significantly across regions. Therefore, local audits of activity and assessment of local needs are critical in addressing specific local demands and will complement these national data. We are still in the early stages of recovery post-COVID-19. Future work should also be directed at seeing how the dynamic rate of recovery evolves, and predictive modelling should be repeated and resource allocation periodically adjusted in response to new trends.

Another avenue for future research is the analysis of emergency ENT services. Emergency procedures constitute a significant component of hospital workload and often influence the availability of resources for elective surgeries. Understanding trends in emergency ENT services and comparing these to elective services will be useful in evaluating their impact on elective care as well as aid in workforce allocation and infrastructure planning.

## 5. Conclusions

This nationwide study shows that, in the UK, the demand for ENT procedures has remained stable over the last decade. However, rhinology procedures demonstrated the most significant upward trend and greatest deficit from COVID-19. The impact of COVID-19 highlighted the vulnerabilities in the current system, with a prolonged recovery period expected. Our findings advocate that ongoing monitoring and strategic planning are essential to ensure the NHS remains responsive to evolving clinical needs.

## Figures and Tables

**Table 1:** Year-by-year COVID-19 backlog breakdown. Derived by calculating the difference between the forecasted levels and the actual volume of procedures.

Year	Otology	Rhinology	Head & Neck
2020–2021	76,034	86,750	37,664
2021–2022	30,330	45,111	10,282
2022–2023	21, 067	37,173	3553
Cumulative backlog	127,431	169,034	51,449

## Data Availability

The data used in this study was obtained from the Hospital Episode Statistics (HES) database, which is openly available through NHS Digital. This is accessible on the following website: https://digital.nhs.uk/data-and-information/data-tools-and-services/data-services/hospital-episode-statistics.

## Supplementary Materials

The following supporting information can be downloaded at: https://www.mdpi.com/article/10.3390/jcm13247850/s1, Supplementary File S1: Table S1: Otology procedures and the OPCS-4 codes included from hospital episode statistics (HES) data; Table S2: Rhinology procedures and OPCS4 codes included from hospital episode statistics (HES) data; Table S3: Head and neck procedures and OPCS4 codes included from hospital episode statistics (HES) data; Supplementary File S2: Trends in the annual volume of otology, rhinology, and head and neck (H&N) procedures in England from 2000–2001 to 2022–2023.
