# Supplementary material for: Evolving Trends and Future Demands in ENT Procedures: A Nationwide 10-Year Analysis
Source: J Clin Med. 2024 Dec 23;13(24):7850. doi: 10.3390/jcm13247850 (PMC11728088; doi:10.3390/jcm13247850)
Supplement: Supplementary file 1 [file jcm-13-07850-s001.zip › supplementary_file S1 JCM.pdf]

Supplementary File S1

Table S1: Otology procedures and OPCS-4 codes included from HES data.

OPCS-4 CODE	PROCEDURE
D01.1	Total excision of external ear
D01.2	Partial excision of external ear
D01.3	Excision of preauricular abnormality
D01.8	Other specified excision of external ear
D01.9	Unspecified excision of external ear
D02.1	Excision of lesion of external ear
D03.1	Reconstruction of external ear using graft
D03.2	Reconstruction of external ear NEC
D03.3	Pinnaplasty
D03.4	Meatoplasty of external ear
D03.8	Other specified plastic operations on external ear
D03.9	Unspecified plastic operations on external ear
D04.1	Drainage of haematoma of external ear
D04.2	Drainage of abscess of external ear
D04.8	Other specified drainage of external ear
D04.9	Unspecified drainage of external ear
D05.5	Placement of hearing implant in external ear
D05.6	Attention to hearing implant in external ear
D05.7	Removal of hearing implant from external ear
D05.8	Other specified attachment of auricular prosthesis
D06.1	Biopsy of lesion of external ear
D06.2	Repair of lobe of external ear
D06.3	Repair of external ear NEC
D06.4	Graft of skin to external ear
D06.5	Flap of skin to external ear
D06.8	Other specified other operations on external ear
D06.9	Unspecified other operations on external ear
D07.1	Irrigation of external auditory canal for removal of wax
D07.2	Removal of wax from external auditory canal NEC
D07.3	Removal of foreign body from external auditory canal
D07.8	Other specified clearance of external auditory canal
D07.9	Unspecified clearance of external auditory canal
D08.1	Extirpation of lesion of external auditory canal
D08.2	Reconstruction of external auditory canal
D08.3	Drainage of external auditory canal
D08.4	Incision of external auditory canal
D08.5	Irrigation of external auditory canal NEC
D08.6	Blind sac closure of external auditory canal
D08.8	Other specified other operations on external auditory canal
D08.9	Unspecified other operations on external auditory canal
D10.1	Radical mastoidectomy NEC
D10.2	Modified radical mastoidectomy
D10.3	Cortical mastoidectomy
D10.4	Simple mastoidectomy

D10.5	Excision of lesion of mastoid
D10.6	Revision of mastoidectomy
D10.8	Other specified exenteration of mastoid air cells
D10.9	Unspecified exenteration of mastoid air cells
D12.1	Obliteration of mastoid
D12.2	Atticotomy
D12.3	Biopsy of mastoid
D12.4	Exploration of mastoid
D12.5	Removal of pack from mastoid
D12.7	Atticoantrostomy
D12.8	Other specified other operations on mastoid
D12.9	Unspecified other operations on mastoid
D13.1	First stage insertion of fixtures for bone anchored hearing prosthesis
D13.2	Second stage insertion of fixtures for bone anchored hearing prosthesis
D13.3	Reduction of soft tissue for bone anchored hearing prosthesis
D13.4	Attention to fixtures for bone anchored hearing prosthesis
D13.5	One stage insertion of fixtures for bone anchored hearing prosthesis
D13.6	Fitting of external hearing prosthesis to bone anchored fixtures
D13.8	Other specified attachment of bone anchored hearing prosthesis
D13.9	Unspecified attachment of bone anchored hearing prosthesis
D14.1	Tympanoplasty using graft
D14.2	Tympanoplasty NEC
D14.3	Revision of tympanoplasty
D14.4	Combined approach tympanoplasty
D14.8	Other specified repair of eardrum
D14.9	Unspecified repair of eardrum
D15.1	Myringotomy with insertion of ventilation tube through tympanic membrane
D15.2	Suction clearance of middle ear
D15.3	Incision of ear drum NEC
D15.8	Other specified drainage of middle ear
D15.9	Unspecified drainage of middle ear
D16.1	Prosthetic replacement of ossicular chain
D16.2	Graft replacement of ossicular chain
D16.8	Other specified reconstruction of ossicular chain
D16.9	Unspecified reconstruction of ossicular chain
D17.1	Stapedectomy
D17.2	Revision of stapedectomy
D17.3	Division of adhesions of ossicle of ear
D17.8	Other specified other operations on ossicle of ear
D17.9	Unspecified other operations on ossicle of ear
D19.1	Excision of lesion of middle ear
D19.2	Destruction of lesion of middle ear
D19.8	Other specified extirpation of lesion of middle ear

D19.9	Unspecified extirpation of lesion of middle ear
D20.1	Biopsy of lesion of middle ear
D20.2	Maintenance of ventilation tube through tympanic membrane
D20.3	Removal of ventilation tube from tympanic membrane
D20.4	Placement of hearing implant in middle ear
D20.5	Attention to hearing implant in middle ear
D20.6	Removal of hearing implant in middle ear
D20.7	Transtympanic injection to middle ear
D20.8	Other specified other operations on middle ear
D20.9	Unspecified other operations on middle ear
D22.1	Graft to eustachian canal
D22.2	Intubation of eustachian canal
D22.3	Insufflation of eustachian canal
D22.8	Other specified operations on eustachian canal
D22.9	Unspecified operations on eustachian canal
D23.1	Transtympanic injection to inner ear
D23.8	Other specified operations on inner ear
D23.9	Unspecified operations on inner ear
D24.1	Implantation of intracochlear prosthesis
D24.2	Implantation of extracochlear prosthesis
D24.3	Attention to cochlear prosthesis
D24.5	Transtympanic electrocochleography
D24.6	Removal of cochlear prosthesis
D24.8	Other specified operations on cochlea
D26.1	Operations on endolymphatic sac
D26.2	Membranous labyrinthectomy
D26.3	Osseous labyrinthectomy
D26.4	Neurectomy of vestibular apparatus
D26.8	Other specified operations on vestibular apparatus
D26.9	Unspecified operations on vestibular apparatus
D28.1	Biopsy of lesion of ear NEC
D28.2	Examination of ear under anaesthetic
D28.8	Other specified other operations on ear
D28.9	Unspecified other operations on ear

Supplementary File S1

Table S2: Rhinology procedures and OPCS-4 codes included from HES data.

OPCS-4 CODE	PROCEDURE
E01.1	Total excision of nose
E01.8	Other specified excision of nose
E01.9	Unspecified excision of nose
E02.1	Total reconstruction of nose
E02.2	Reconstruction of nose NEC
E02.3	Septorhinoplasty using implant
E02.4	Septorhinoplasty using graft
E02.5	Reduction rhinoplasty
E02.6	Rhinoplasty NEC
E02.7	Alar reconstruction with cartilage graft
E02.8	Other specified plastic operations on nose
E02.9	Unspecified plastic operations on nose
E03.1	Submucous excision of septum of nose
E03.2	Excision of lesion of septum of nose
E03.3	Biopsy of lesion of septum of nose
E03.4	Closure of perforation of septum of nose NEC
E03.5	Incision of septum of nose
E03.6	Septoplasty of nose NEC
E03.7	Septal reconstruction with cartilage graft
E03.8	Other specified operations on septum of nose
E03.9	Unspecified operations on septum of nose
E04.1	Submucous diathermy to turbinate of nose
E04.2	Excision of turbinate of nose NEC
E04.3	Excision of lesion of turbinate of nose NEC
E04.4	Division of adhesions of turbinate of nose
E04.5	Biopsy of lesion of turbinate of nose
E04.6	Cauterisation of turbinate of nose
E04.7	Surgical outfracture of turbinate of nose
E04.8	Other specified operations on turbinate of nose
E04.9	Unspecified operations on turbinate of nose
E05.1	Cauterisation of internal nose
E05.2	Ligation of artery of internal nose
E05.3	Embolisation of artery of internal nose
E05.4	Laser therapy of internal nose
E05.8	Other specified surgical arrest of bleeding from internal nose
E05.9	Unspecified surgical arrest of bleeding from internal nose
E06.1	Packing of posterior cavity of nose NEC
E06.2	Packing of anterior cavity of nose NEC
E06.3	Removal of packing from cavity of nose
E06.4	Balloon packing of cavity of nose
E06.8	Other specified packing of cavity of nose
E06.9	Unspecified packing of cavity of nose
E07.1	Correction of stenosis of nasal pyriform aperture
E07.2	Septodermoplasty

E07.3	Septorhinoplasty NEC
E07.8	Other specified other plastic operations on nose
E08.1	Polypectomy of internal nose
E08.2	Extirpation of lesion of internal nose NEC
E08.3	Correction of congenital atresia of choana
E08.4	Division of adhesions of internal nose
E08.5	Removal of foreign body from cavity of nose
E08.6	Surgical closure of anterior nares
E08.7	Surgical reopening of anterior nares
E08.8	Other specified other operations on internal nose
E08.9	Unspecified other operations on internal nose
E09.1	Excision of lesion of external nose
E09.2	Destruction of lesion of external nose NEC
E09.3	Suture of external nose
E09.4	Shave of skin of nose
E09.5	Biopsy of lesion of external nose
E09.6	Laser destruction of lesion of external nose
E09.7	Graft of skin to external nose
E09.8	Other specified operations on external nose
E09.9	Unspecified operations on external nose
E10.1	Biopsy of lesion of nose NEC
E10.8	Other specified other operations on nose
E10.9	Unspecified other operations on nose
E11.1	One stage attachment of fixtures for nasal prosthesis NEC
E11.2	First stage attachment of fixtures for nasal prosthesis
E11.3	Second stage attachment of fixtures for nasal prosthesis
E11.4	Revision of fixtures for attachment of nasal prosthesis
E11.5	Removal of fixtures for attachment of nasal prosthesis
E11.6	Attachment of nasal prosthesis
E11.8	Other specified operations on fixtures for nasal prosthesis
E11.9	Unspecified operations on fixtures for nasal prosthesis
E12.1	Ligation of maxillary artery using sublabial approach
E12.2	Drainage of maxillary antrum using sublabial approach
E12.3	Irrigation of maxillary antrum using sublabial approach
E12.4	Transantral neurectomy of vidian nerve using sublabial approach
E12.8	Other specified operations on maxillary antrum using sublabial approach
E13.1	Drainage of maxillary antrum NEC
E13.2	Excision of lesion of maxillary antrum
E13.3	Intranasal antrostomy
E13.4	Biopsy of lesion of maxillary antrum
E13.5	Closure of fistula between maxillary antrum and mouth
E13.6	Puncture of maxillary antrum
E13.7	Neurectomy of vidian nerve NEC
E13.8	Other specified other operations on maxillary antrum
E13.9	Unspecified other operations on maxillary antrum
E14.1	External frontoethmoidectomy

E14.2	Intranasal ethmoidectomy
E14.3	External ethmoidectomy
E14.4	Transantral ethmoidectomy
E14.5	Bone flap to frontal sinus
E14.6	Trephine of frontal sinus
E14.7	Median drainage of frontal sinus
E14.8	Other specified operations on frontal sinus
E14.9	Unspecified operations on frontal sinus
E15.1	Drainage of sphenoid sinus
E15.2	Puncture of sphenoid sinus
E15.3	Repair of sphenoidal sinus
E15.4	Excision of lesion of sphenoid sinus
E15.8	Other specified operations on sphenoid sinus
E15.9	Unspecified operations on sphenoid sinus
E16.1	Frontal sinus osteoplasty
E16.2	Drainage of frontal sinus NEC
E16.8	Other specified other operations on frontal sinus
E16.9	Unspecified other operations on frontal sinus
E17.1	Excision of nasal sinus NEC
E17.2	Excision of lesion of nasal sinus NEC
E17.3	Biopsy of lesion of nasal sinus NEC
E17.4	Lateral rhinotomy into nasal sinus NEC
E17.8	Other specified operations on unspecified nasal sinus
E17.9	Unspecified operations on unspecified nasal sinus

Table S3: Head and Neck Procedures and OPCS-4 codes included from HES data.

OPCS-4 CODE	PROCEDURE
E19.1	Total pharyngectomy
E19.2	Partial pharyngectomy
E19.8	Other specified excision of pharynx
E19.9	Unspecified excision of pharynx
E20.1	Total adenoidectomy
E20.2	Biopsy of adenoid
E20.3	Surgical arrest of postoperative bleeding of adenoid
E20.4	Suction diathermy adenoidectomy
E20.8	Other specified operations on adenoid
E20.9	Unspecified operations on adenoid
E21.1	Pharyngoplasty using posterior pharyngeal implant
E21.2	Pharyngoplasty using posterior pharyngeal flap
E21.3	Pharyngoplasty using lateral pharyngeal flap
E21.4	Plastic repair of pharynx NEC
E21.8	Other specified repair of pharynx
E21.9	Unspecified repair of pharynx
E23.1	Open excision of lesion of pharynx
E23.2	Operations on pharyngeal pouch
E23.8	Other specified other open operations on pharynx
E23.9	Unspecified other open operations on pharynx
E24.1	Endoscopic extirpation of lesion of nasopharynx
E24.2	Endoscopic extirpation of lesion of pharynx NEC
E24.3	Endoscopic operations on pharyngeal pouch
E24.8	Other specified therapeutic endoscopic operations on pharynx
E24.9	Unspecified therapeutic endoscopic operations on pharynx
E25.1	Diagnostic endoscopic examination of nasopharynx and biopsy of lesion of nasopharynx
E25.2	Diagnostic endoscopic examination of pharynx and biopsy of lesion of pharynx NEC
E25.3	Diagnostic endoscopic examination of nasopharynx NEC
E25.8	Other specified diagnostic endoscopic examination of pharynx
E25.9	Unspecified diagnostic endoscopic examination of pharynx
E27.1	Open biopsy of lesion of pharynx
E27.2	Drainage of retropharyngeal abscess
E27.3	Incision of pharynx NEC
E27.4	Removal of foreign body from pharynx
E27.5	Dilation of pharynx
E27.6	Examination of pharynx under anaesthetic
E27.8	Other specified other operations on pharynx
E27.9	Unspecified other operations on pharynx
E28.1	Cricopharyngeal myotomy
E28.8	Other specified operations on cricopharyngeus muscle

E28.9	Unspecified operations on cricopharyngeus muscle
E29.1	Total laryngectomy
E29.2	Partial horizontal laryngectomy
E29.3	Partial vertical laryngectomy
E29.4	Partial laryngectomy NEC
E29.5	Laryngofissure and cordectomy of vocal cord
E29.6	Laryngectomy NEC
E29.7	Cordectomy of vocal cord NEC
E29.8	Other specified excision of larynx
E29.9	Unspecified excision of larynx
E30.1	Excision of lesion of larynx using thyrotomy as approach
E30.2	Excision of lesion of larynx using lateral pharyngotomy as approach
E30.3	Open destruction of lesion of larynx
E30.8	Other specified open extirpation of lesion of larynx
E30.9	Unspecified open extirpation of lesion of larynx
E31.1	Laryngotracheal reconstruction using cartilage graft
E31.2	Laryngotracheoplasty NEC
E31.3	Division of stenosis of larynx and insertion of prosthesis into larynx
E31.4	Implantation of artificial voice box into larynx
E31.5	Attention to artificial voice box in larynx
E31.8	Other specified reconstruction of larynx
E31.9	Unspecified reconstruction of larynx
E33.1	External arytenoidectomy
E33.2	Cordopexy of vocal cord
E33.3	Operations on cartilage of larynx NEC
E33.4	Open biopsy of lesion of larynx
E33.5	Vocal cord medialisation using implant
E33.6	Vocal cord medialisation using biological material
E33.8	Other specified other open operations on larynx
E33.9	Unspecified other open operations on larynx
E34.1	Microtherapeutic endoscopic extirpation of lesion of larynx using laser
E34.2	Microtherapeutic endoscopic resection of lesion of larynx NEC
E34.3	Microtherapeutic endoscopic destruction of lesion of larynx NEC
E34.8	Other specified microtherapeutic endoscopic operations on larynx
E34.9	Unspecified microtherapeutic endoscopic operations on larynx
E35.1	Endoscopic arytenoidectomy
E35.2	Endoscopic resection of lesion of larynx
E35.3	Endoscopic destruction of lesion of larynx
E35.4	Endoscopic removal of prosthesis from larynx
E35.5	Endoscopic removal of foreign body from larynx

E35.6	Endoscopic partial laryngectomy
E35.7	Endoscopic vocal cord medialisation
E35.8	Other specified other therapeutic endoscopic operations on larynx
E35.9	Unspecified other therapeutic endoscopic operations on larynx
E36.1	Diagnostic endoscopic examination of larynx and biopsy of lesion of larynx
E36.8	Other specified diagnostic endoscopic examination of larynx
E36.9	Unspecified diagnostic endoscopic examination of larynx
E37.1	Diagnostic microendoscopic examination of larynx and biopsy of lesion of larynx
E37.8	Other specified diagnostic microendoscopic examination of larynx
E37.9	Unspecified diagnostic microendoscopic examination of larynx
E38.1	Injection into larynx
E38.8	Other specified other operations on larynx
E38.9	Unspecified other operations on larynx
E39.1	Open excision of lesion of trachea
E39.8	Other specified partial excision of trachea
E39.9	Unspecified partial excision of trachea
E40.1	Reconstruction of trachea and anastomosis HFQ
E40.2	Reconstruction of trachea using graft
E40.3	Reconstruction of trachea NEC
E40.8	Other specified plastic operations on trachea
E41.1	Open insertion of tubal prosthesis in trachea
E41.2	Open renewal of tubal prosthesis in trachea
E41.3	Open removal of tubal prosthesis from trachea
E41.4	Tracheo-oesophageal puncture with insertion of speech prosthesis
E41.8	Other specified open placement of prosthesis in trachea
E41.9	Unspecified open placement of prosthesis in trachea
E42.1	Permanent tracheostomy
E42.2	Cricothyroidostomy
E42.3	Temporary tracheostomy
E42.4	Revision of tracheostomy
E42.5	Closure of tracheostomy
E42.6	Replacement of tracheostomy tube
E42.7	Removal of tracheostomy tube
E42.8	Other specified exteriorisation of trachea
E42.9	Unspecified exteriorisation of trachea
E43.1	Open destruction of lesion of trachea
E43.2	Tracheorrhaphy
E43.3	Tracheopexy
E43.4	Open biopsy of lesion of trachea
E43.5	Closure of tracheocutaneous fistula

E43.8	Other specified other open operations on trachea
E43.9	Unspecified other open operations on trachea
