# Supplementary figures and images for: Evolving Trends and Future Demands in ENT Procedures: A Nationwide 10-Year Analysis
Source: J Clin Med. 2024 Dec 23;13(24):7850. doi: 10.3390/jcm13247850 (PMC11728088; doi:10.3390/jcm13247850)

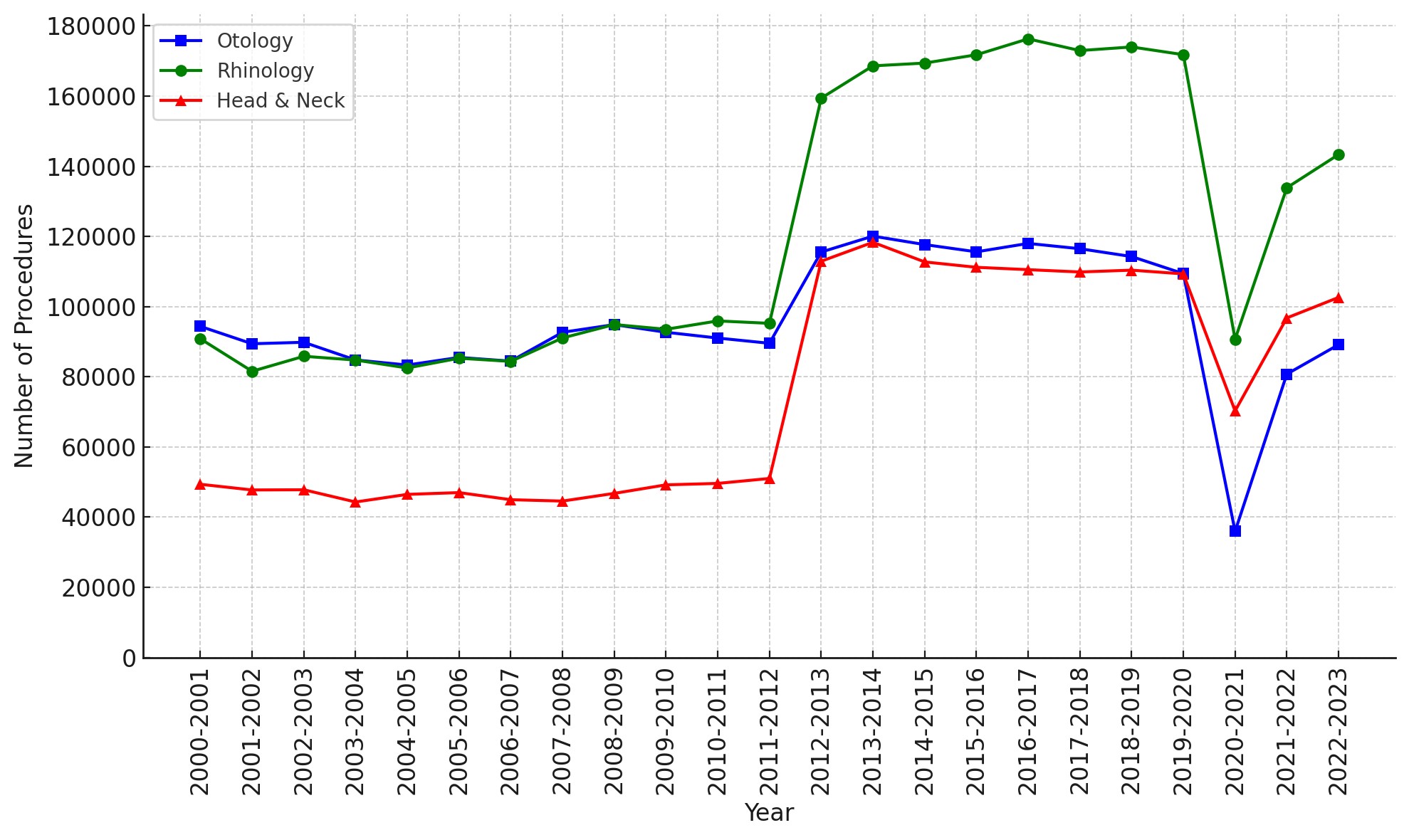

Supplement: Supplementary file 1 [file jcm-13-07850-s001.zip › supplementary_file S2 JCM.jpeg]
